# Drug-like Properties and Fraction Lipophilicity Index as a combined metric

**DOI:** 10.5599/admet.1022

**Published:** 2021-10-10

**Authors:** Anna Tsantili-Kakoulidou, Vassilis J. Demopoulos

**Affiliations:** 1 Department of Pharmaceutical Chemistry, School of Pharmacy, National and Kapodistrian University of Athens, Panepistimiopolis, Zografou, 157 71 Athens, Greece. E-mail: tsantili@pharm.uoa.gr; 2 Department of Pharmaceutical Chemistry, School of Pharmacy, Aristotle University of Thessaloniki, 54124 Thessaloniki, Greece. E-mail: vdem@pharm.auth.gr

**Keywords:** drug-likeness, oral drugs, Fraction Lipophilicity Index, Rule of 5, Molecular weight, Polar atoms

## Abstract

Fraction Lipophicity Index (FLI) has been developed as a composite drug-like metric combining log *P* and log *D* in a weighted manner. In the present study, an extended data set confirmed the previously established drug-like FLI range 0-8 using two calculation systems for log *P*/log *D* assessment, the freeware MedChem Designer and ClogP. The dataset was split into two classes according to the percentage of fraction absorbed (%FA) - class 1 including drugs with high to medium absorption levels and class 2 including poorly absorbed drugs. The FLI and FLI-C (ClogP based FLI) drug-like range covers 92 % and 91 % of class 1 drugs, respectively. Using MlogP, a narrower drug-like FLI-M range 0-7 was established, covering 91 % of class 1 drugs. The dependence of the degree of ionization to intrinsic lipophilicity within the FLI (FLI-C, FLI-M) drug-like range as well as the inter-relation between the other Ro5 properties (Mw, HD, HA) was explored to define drug-like / non-drug-like combinations as a safer alternative to single properties for drug candidates’ prioritization. In this sense, we propose a combined metric of Mw and the number of polar atoms (Mw/NO) to account for both size and polarity. Setting the value 50 as cutoff, a distinct differentiation between class 1 and class 2 drugs was obtained with Mw/NO>50 for more than 70 % of class 1 drugs, while the opposite was observed for class 2 drugs.

## Introduction

The role of physicochemical properties in controlling the fate of drug molecules within the organism and their binding to macromolecules has been well documented. Emphasis has been given to lipophilicity, molecular size and polarity as the most crucial properties, which have been incorporated in the majority of the metrics used to set boundaries in the drug-like chemical space [1,2]. There is strong evidence that compounds with higher lipophilicity and higher molecular weight, *e.g*., with high molecular ‘obesity’ are more likely to be discontinued during clinical development [3-6]. They are associated with difficulties in oral absorption, while they are considered to exhibit increased ‘promiscuity’ towards biomacromolecules. As a result, they may lack selectivity, leading to side effects while they accumulate in the organism, increasing non-selective toxicity. The minimal hydrophobicity concept was formulated in 1987 by Hansch et al. [7], who suggested that molecules exert their action rather by specific binding and not by non-specific hydrophobic interactions. Ten years later, the well-known Rule of Five (Ro5) formulated by Lipinski for compounds intended for oral administration [8] suggested upper limits for lipophilicity (clog*P*≤5), molecular weight (*M*_W_≤500), counts of hydrogen bond donor (HD≤5) and acceptor sites (HA≤10), the latter two expressing polarity. Compounds violating more than two of the Ro5 conditions are prone to gastrointestinal absorption problems. Ro5 as well as limits or ranges for additional drug-like properties such as a number of rotatable bonds, polar surface area, or a number of aromatic rings [2,9-11] may be used as virtual screening filters in early drug discovery. However, this strategy has been recently disputed, as there is an increasing interest in exploring beyond the Rule of five (bRo5) chemical space in drug discovery [12,13].

The concept of drug-likeness was further advanced to address drug safety by normalizing affinity to size or/and lipophilicity, as reflected in metrics like Ligand Efficiency (LE), Lipophilic Ligand Efficiency (LLE) and Ligand Efficiency Dependent Lipophilicity (LELP) [1,14-16]. On the other hand, Ghose et al. [17] suggest qualifying ranges of -0.4 to 5.6 for log *P* and 160 to 480 for molecular weight as filters for drug-likeness, thus setting lower limits lipophilicity and size [17].

In the above metrics, lipophilicity is expressed by log *P*, which corresponds to intrinsic lipophilicity of the neutral species, ignoring the effect of ionization in the case of drugs containing ionizable groups. Intrinsic lipophilicity governs hydrophobic binding to biomacromolecules. However, permeability is affected rather by apparent lipophilicity, log *D*, as dictated by the partition-pH hypothesis, although intrinsic lipophilicity remains the driving force. In a retrospective analysis of human bioavailability data, compounds with log *D* values at pH 6.5 in the range of -2 to 3 were found to display increased bioavailability [18]. For physiological pH, an optimum log *D* range between 1-3 has been proposed [19], while Waring established lower limits for log *D*_7.4_ in a molecular weight-dependent manner to achieve > 50 % chance for high permeability [20]. According to the author, the log *D* thresholds occur at higher values for higher molecular weight ranges, with large molecules (*M*w>500) necessitating log *D*> 4.5. Analogous results on the dependence of optimal log *D* ranges on molecular weight were also reported by other authors [21]. The above suggestions underestimate the role of intrinsic lipophilicity, although the dependence of log *D* to molecular weight in Waring’s approach indirectly introduces the bulk component, which is related to the hydrophobicity of the molecule, according to the dual nature of lipophilicity [22]. Moreover, since log *D* values depend on log *P*, their magnitude alone does not provide information of the degree of ionization. A specific log *D* value may correspond to log *P* values spanning within 3 log units, considering that this is the difference between log *P* of the neutral and fully ionized species.

Recently, we have developed a new metric for assessing oral drug-likeness of ionizable chemical entities as a weighted combination of both log *P* and log *D*, considering that the two measures have a distinct role to play [23,24]. We refer to this metric as Fraction Lipophilicity Index (FLI). FLI is defined as the logarithm of the quotient of partition coefficients (*P*) and the fraction of the neutral form (*f*^N^) at a given pH (i.e., log(*P*/*f*^N^)), which upon analyzing log *f*^N^ leads to equation 1:


(1)

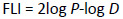



Eq.1 was further modified to eq. 2 by considering the absolute value of log *D.* This modification was assumed necessary since negative log *D* values, although unfavorable for absorption, contribute positively to FLI calculation shifting the values to the center of the FLI range.


(2)

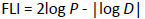



The drug-like FLI range 0-8, based on calculations with the MedChem Designer freeware, was found to accommodate 89 % of drugs classified as highly or moderately absorbed, with the range 5 to 6 being the most populated. There is a slight shift of FLI distribution to the upper limits of the above range for acidic compounds, which is restored if FLI@pH5.5 is considered. This is justified assuming that absorption of acids is favored in the upper part of the intestine. The same drug-like FLI range was found to be covered by 73 % of drugs with low absorption, while FLI distribution is shifted towards negative values.

The present study is a continuation of our previous investigation [23,24] in the aim to further elaborate the FLI metric, extending the data set to include drugs approved till December 2020, as well as a larger number of older drugs, while using two additional log *P* calculation systems, namely clog*P* for direct comparison with Lipinski’s Ro5 and Mlog*P* for which an upper limit 4.15 is suggested. The dependence of the degree of ionization to intrinsic lipophilicity within the drug-like space and the inter-relation between the Ro5 properties are explored to define drug-like / non-drug-like combinations as a safer alternative to single properties for drug candidates’ prioritization, integrating FLI in this perspective.

## Materials and methods

The entire dataset comprises 643 drugs, presented in table 1S, in Supplementary Material along with their SMILES. It includes all drugs analyzed in our previous publication [24], drugs compiled by Newby et al. [25], and later generation drugs approved in 2017-2020. All drugs included in the data set possess ionizable centers and display a degree of ionization at pH 7.4 for bases and pH 5.5 for acids higher than 30 %, corresponding to a difference Δ(log *P*-log *D*) > 0.14. Depending on their isoelectric point, zwitterions were considered if log *D*_max_ > 0.14 than log *D* at pH 7.4 or pH 5.5.

The dataset is split into two classes according to their fraction absorbed (%FA) taken from Newby’s compilation [25]. Fraction absorbed was considered more appropriate than bioavailability, used in our previous publications, since the latter is a composite parameter involving several factors, such as gastrointestinal absorption, chemical stability, and the first-pass effect. For classification to class 1 (highly and medium absorbed drugs) and class 2 (poorly absorbed drugs), the cutoff value was set at 45 % FA. Drugs in class 1 are designated with ‘M’ if %FA is in the range of 46-60 and with ‘H’ if %FA is higher than 60. Drugs in class 2 are designated with ‘L’. For drugs with not available %FA in ref [25], information on absorption was taken from DrugBank or other sources, as presented in Table 1S, Supplementary Material. Moreover, latest generation drugs with not available %FA data have been classified into class 1, if they are administered orally or to class 2 if other route of administration is reported. Class 1 comprises 527 drugs (336 bases, 178 acids and 13 zwitterions) with high or moderate absorption, while class 2 comprises 116 drugs (54 bases, 54 acids and 8 zwitterions).

### Calculation of FLI

Partition coefficients (logPS^+^) and distribution coefficients at physiological pH for bases and pH 5.5 for acids (logDS^+^) were calculated using the freeware MedChem Designer(TM) version 3.0.0.28 (https://www.simulations-plus.com/). The same program provides log *P* calculations according to Moriguchi (MlogP) [26]. For direct comparison with Ro5, the software ClogP for Windows v.4.0 [http://www.biobyte.com/] was also used (clogP). MlogD and ClogD values were assessed considering the difference Δ(log *P –* log *D*) generated by MedChem Designer. Δ(log *P* – log *D*) corresponds to [log(10^±pH-pKa)^+1] and is independent of the log *P* calculation system. In the expression Δ(log *P* – log *D*), log *D* stands for log *D*_7.4_ and log *D*_5.5_ for bases and acids, respectively. The program did not provide calculation of clogP for five compounds with high *M*w (>1000). For these cases, clogP values were predicted using eq. 3 which reflects the correlation between clogP and logPS^+^ values.


(3)

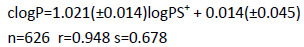



Equation 3 practically corresponds to 1:1 correlation. The mean difference Δ(clogP - log PS^+^) is 0.07, although larger deviations are observed in individual cases (Table 1S, Supplementary Material). Eq. 3 was generated upon exclusion of 16 drugs with a high difference, |Δ(clogP - logPS^+^)| > 2.

All lipophilicity data are presented in Table 1S, Supplementary Material. In the same Table, molecular weight (*M*w), number of hydrogen bond donor (HD) and acceptor sites (HA), designated as [N+O], as well as Ro5 violation scores, are provided. *M*w, HD and [N+O] were calculated by MedChem Designer. Ro5 are labeled according to log *P* calculation systems as scoreRo5/logPS^+^, scoreRo5/clogP and scoreRo5/MlogP, respectively. Calculations of Ro5 scores have been performed manually for the first two, while MedChem Designer has provided scoreRo5/MlogP. FLI values were calculated according to eq. 2 for pH=7.4 for bases and pH=5.5 for acids. FLI values of zwitterions were calculated either at pH 7.4 or at pH 5.5 according to their isoelectric point. FLI-C and FLI-M were calculated using the corresponding clogP/clogD and MlogP/MlogD values. FLI, FLI-C and FLI-M are included in Table 1S, Supplementary Material.

### Statistical analysis

The program STATISTICA 7.1, Copyright© StatSoft Inc. 1984-2006, 2300 East 14th Street, Tulsa, OK 74104, USA was used for statistical analysis and histograms’ construction.

## Results and Discussion

### Data overview

The property overview of the drugs considered in the present study is shown in Table 1 (Supplementary tables). Mean logPS^*+*^, clogP and MlogP values are 2.63, 2.68 and 2.02, respectively, while mean *M*w is 372.5. Clear differentiation in the mean values is observed between class 1 and 2 drugs. Class 1 drugs display higher mean lipophilicity and lower mean *M*w, while the opposite is found for class 2. Moreover, class 2 drugs possess a considerably higher number of hydrogen bond donor (HD) and acceptor sites ([N+O]). For class 1 drugs, high *M*w is associated with polar atoms [N + O] up to 15, while in class 2, large molecules possess up to 33 polar atoms and lower log *P* values.

It should be noted that mean log *P* and *M*w values for class 1 are considerably higher than those reported for 1791 oral drugs of the ChEMBL database -including both neutral and ionizable molecules- which are close to 2.5 and 333, respectively [27,28]. As also commented in our previous publication [24], there is a shift towards higher log *P* and *M*w for drugs approved after 2000, which display mean logPS^+^/clogP and *M*w values 3.34/3.42 and 430.9 respectively. In particular, for drugs approved between 2000-2009 mean logPS^+^/clogP/MlogP and *M*w are 3.39/3.38/2.71 and 365.1, respectively, while a further increase in mean *M*w to 465.1 is observed for drugs approved in the last decade (2010-2020). For drugs before 2000, descriptive statistics show mean logPS^+^ clogP and MlogP 2.40, 2.44 and 1.88 respectively and a mean *M*w equal to 353.5. More to the point, a tendency to increase the number of polar atoms is evident after 2000 (Table 1, Supplementary tables). Analogous findings are reported by Hann and Keserü [29] and Shulz [30], who outlined property differences between target classes and companies, suggesting apparently different requirements of the expanding target space. This is, for instance, the case of PPAR- γ agonists [31] or protease inhibitors [32], indicating that drug-like properties should be adapted over time.

### Exploring drug-like properties inter-relation

**Figure 1.**
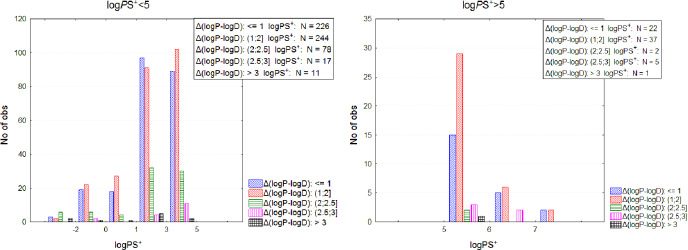
Ionization pattern for drugs with logPS^+^ ≤5 (left) and logPS^+^ >5 (right)

As aforementioned, log *D* values per se do not give information about ionization if log *P* is not considered. To explore ionization throughout the logPS^+^ range, the difference Δ(log *P* – log *D*) was compared to two logPS^+^ levels, above and below the drug-like limit of five. It was found that maximum Δ(log *P* – log *D*), e.g. maximum ionization, is differentiated between the two logPS^+^ levels in both class 1 and class 2, with reduced ionization for highly lipophilic drugs (Table 2, Supplementary tables). The ionization pattern according to logPS^+^ boundaries is provided in Figure 1. The log *P* dependent ionization will be further discussed in relation to the FLI concept.

**Figure 2.**
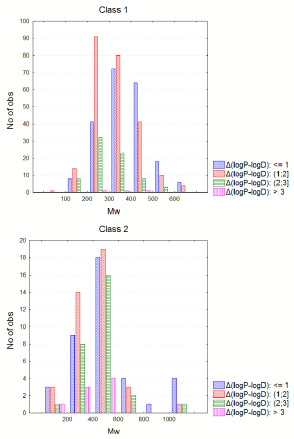
*M*w dependent ionization levels for class 1 and class 2. (X-axis is in different scale to avoid compressing of the histograms in class 1, which does not contain drugs with very high molecular weight, as it is the case for class 2 drugs).

For class 1 drugs, ionization is observed to be also size-dependent. Setting a discriminant at *M*w=600 reduced ionization is observed for drugs with *M*w>600 with maximum Δ(log *P* – log *D*) equal to 1.75, while for drugs with *M*w<600, maximum Δ(log *P* – log *D*) is 3.49. The analogous situation is observed for class 2 drugs, although higher ionization is generally observed with maximum Δ(log *P* – log *D*) values 2.31 and 4.89 for *M*w>600 and *M*w<600, respectively (Table 2, Supplementary tables). Figure 2 shows the different ionization levels according to *M*w for class 1 and class 2 separately.

**Figure 3.**
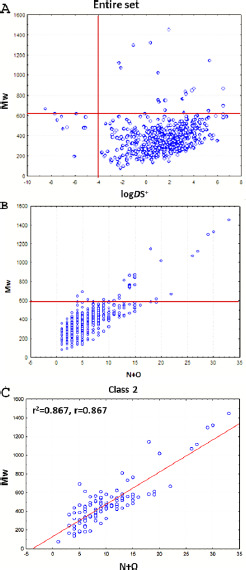
**A**: Plot of Mw versus logPS^+^ and logDS^+^ for the entire set. **B,C**: Plot of Mw versus the number of polar atoms [N+O] for the entire set and class 2 drugs respectively.

Regarding log *D*, there is a tendency to increase with *M*w. However, this trend is not followed for drugs with *M*w>600 and very hydrophilic drugs (Figure 3A). For large molecules, this is due to a higher number of polar atoms [N + O], which contribute negatively to log *P* and, thereupon to log *D* values (Figure 3B). In particular, for class 2 drugs, there is a correlation with r=0.860 between *M*w and the number of polar atoms [N+O] (Figure 3C).

### Drugs beyond the drug-like limits

**Figure 4.**
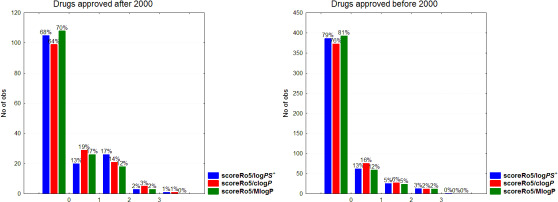
Number of polar atoms [N+O] according to *M*w levels for class 1 and class 2 drugs.

**Figure 5.**
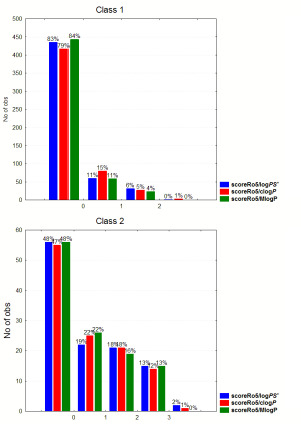
Distribution of Ro5 violations in class 1 and class 2 drugs based on logPS^+^ and clogP.

Recently, there has been an increased interest in the development of drugs beyond the Rule of five (bRo5), especially in the area of oncology and direct-acting antivirals (DAA) [13]. In this aspect we explored violations of Ro5 based on logPS^+^ (scoreRo5/logPS^+^), clogP (scoreRo5/clogP) and MlogP (scoreRo5/MlogP) classifying drugs to those approved after or before 2000. As shown in Figure 4, there is a considerable increase in the percentage of drugs with more than two violations after 2000 with 20, 18 and 14 % according to logPS^+^, clog*P* and Mlog*P* systems, respectively, compared to the corresponding 8, 8 and 7 % before 2000 (Figure 4). If we focus on class 1 drugs approved after 2000, the bRo5 cases are 16, 15 and 10 %, respectively (Figure not shown). Nevertheless, there is still a considerable differentiation in the distribution of Ro5 violations between class 1 and class 2 drugs. For class 1, 82, 79 and 84 % of the drugs show 0 violations, respectively, 11,15 and 11 % display one violation and only 6, 5 and 4 % have higher than two violations. For class 2 drugs, zero violation is displayed for 48, 47 and 48 %, respectively, while 33, 31 and 29 % showed higher than 2. In Figure 5 the distribution of violations in classes 1 and 2 are displayed as bars (left), and in the case of scoreRo5/logPS^+^ as pies (right).

Violations are presented in detail and compared to the corresponding %FA levels in Table 3 (Suppl. Tables), also considering low lipophilicity limits, namely log *P* ≤ -0.4, the minimum value suggested by Ghose et al. [17] and log *P* ≤ -1, set in this paper as a more determinative limit for oral absorption. 47 drugs display logPS^+^<-0.4, 27 out of them with additional violations of the Ro5 drug-like limits. Eleven drugs (55 %) out of the 20 with no other Ro5 violations and 20 drugs with additional Ro5 violations (74 %) have low absorption. If the cutoff value is set to logPS^+^< -1, the percentage of low %FA increases to 66.7 % (8 out of 12 drugs) and 82 % (14 out of 17) for drugs without additional Ro5 violations. Looking at the upper extreme, 67 drugs have logPS^+^>5. Among them, only eight drugs (12 %) have low %FA. Considering the total population of class 2, 33 drugs (28 %) are more hydrophilic (logPS^+^<-0.4), while only 8 (6.9 %) exceed the Ro5 log *P* limit, indicating that high lipophilicity is rather overrated as an issue for oral absorption.

Regarding molecular weight, 80 drugs exceed the limit value of 500, 39 out of them (48.7 %) showing low %FA. Among 20 drugs with twofold logPS^+^/*M*w violations, four (20 %) have low absorption. On the other hand, among the 13 drugs with logPS^+^<-0.4 and *M*w>500, 12 (92 %) and all ten drugs with logPS^+^<-1 and *M*w >500 display low absorption.

An analogous pattern is observed if clogP or MlogP is used instead of logPS^+^ (Table 3, Supplementary tables), although the cases are not always overlapping. These findings indicate that high *M*w in combination with polarity is more crucial than high lipophilicity for absorption difficulties. More to the point, considering hydrogen bonding potential, among the 22 drugs with violations of both hydrogen bond donor and acceptor sites (HD+[N+O]) 20 (91 %) show low absorption.

### Fraction Lipophilicity Index (FLI)

In our previous investigation [24] the drug-like FLI range 0-8 has been suggested, covering 89 % of the drugs with satisfactory absorption. This range was found to cover 73 % of the drugs with lower absorption, with the distribution shifted towards negative FLI values.

**Figure 6.**
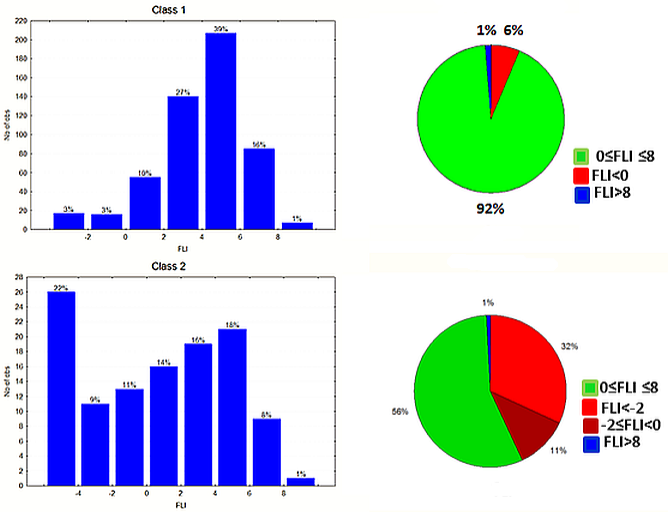
FLI distribution for class 1 and class 2 drugs as histograms (left) and pie charts (right).

**Figure 7.**
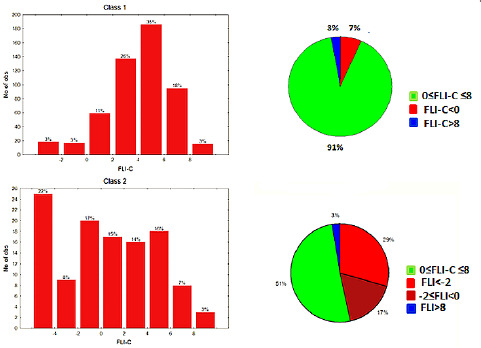
FLI-C distribution for class 1 and class 2 drugs as histograms (left) and pie charts (right).

**Figure 8.**
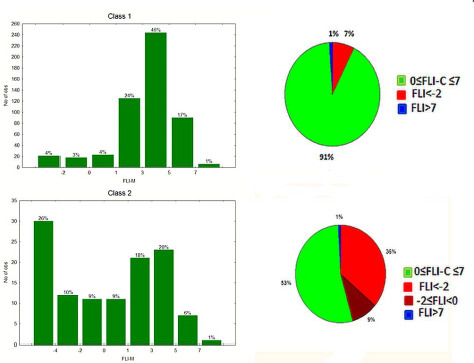
FLI-M distribution for class 1 and class 2 drugs as histograms (left) and pie charts (right).

The above findings were further supported by the extended data set in the present study, with 92 % of class 1 drugs lying within the FLI range 0-8. 6 % have negative FLI values and 1 % FLI values > 8. Most populated is the FLI range 2-6, covering 66 % of the drugs. For class 2 drugs the FLI range 0-8 is covered by 56 % of the drugs and 34 % are within the range 2-6. 1 % have FLI values >8, while 42 % have negative FLI values (Figure 6). The substantially lower coverage of the drug-like FLI range by class 2 drugs, compared to our previous report [24], should be attributed to the use of %FA in this study instead of the more complex bioavailability. Analogous results are obtained using FLI-C, which is based on clogP. The FLI-C drug-like range 0-8 is covered by 91 % of class 1 drugs, 61 % being within the range 2-6. For class 2 the range 0-8 is covered by 51 % of the drugs, while 46 % have negative FLI-C values (Figure 7). Based on the relatively lower MlogP values, FLI-M shapes a narrower drug-like range between 0-7, covering 91 % of class 1 drugs with most populated the region 3-5 (46 %). For class 2 drugs, 53 % are within the range 0-7 and 45 % have negative values (Figure 8).

It is evident that FLI, FLI-C and FLI-M based on different calculation systems show the same performance, although in FLI-M the drug-like range is compressed, in agreement with the lower MlogP upper limit. Thus, in the next section, FLI and FLI-M are considered for further discussion.

### FLI as a combined metric - comparison with Ro5

**Figure 9.**
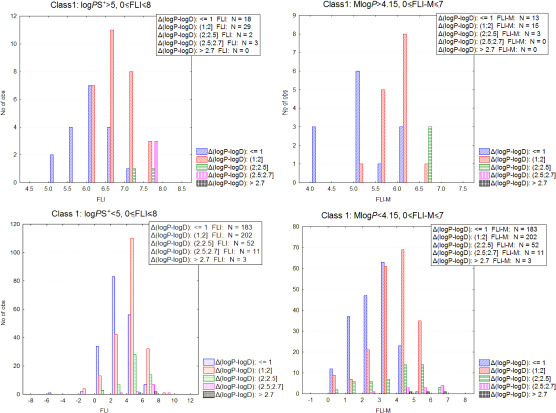
**Left**: Ionization pattern for class 1 drugs, according to FLI levels within the drug-like range, for subsets including drugs with logPS^+^>5 and logPS^+^≤5 respectively. **Right**: Ionization pattern for class 1 drugs, according to FLI-M levels within the drug-like range, for subsets including drugs with logPS^+^>4.15 and logPS^+^≤4.15 respectively.

Considering ionizable drugs, the drug-like FLI range 0-8 expands the lipophilicity cutoff values beyond 5 (or 4.15 for MlogP). In fact, log *P* values (logPS^+^ or clogP) expand to 7.7, provided that the compounds do not exhibit higher ionization than 50 % (considering that at 50 % ionization, lipophilicity decreases by 0.3 log units) or to slightly higher (7.87) if 30 % ionization is considered. For MlogP the upper limits expand to 6.7 or 6.87. Such hard limit values are not realistic for the design of new drugs. Our proposition is to think in terms of combinations of properties rather than single properties. In this aspect, FLI represents a weighted combination of log *P* with log *D*, suggesting reduced ionization for drugs at the upper lipophilicity extremes. On the other hand, low log *P* and log *D* values leading to negative FLI values are more discriminating for low absorption levels. In Figure 9, the FLI values inside the drug-like range along with the associated Δ(log *P* – log *D*) levels for class 1 drugs are presented. For drugs with logPS^+^>5 Δ(log *P* – log *D*) values do not exceed 2.7, with most drugs showing Δ(log *P* – log *D*)<2. Although the majority for class 1 drugs with logPS^+^≤5 has also Δ(log *P* – log *D*)<2, 67 drugs show higher ionization. Similarly, no class 1 drugs with MlogP > 4.15 have Δ(log *P* – log *D*)>2.5, while for 14 drugs with MlogP≤4.15 Δ(log *P* – log *D*) exceeds 2.5.

**Figure 10.**
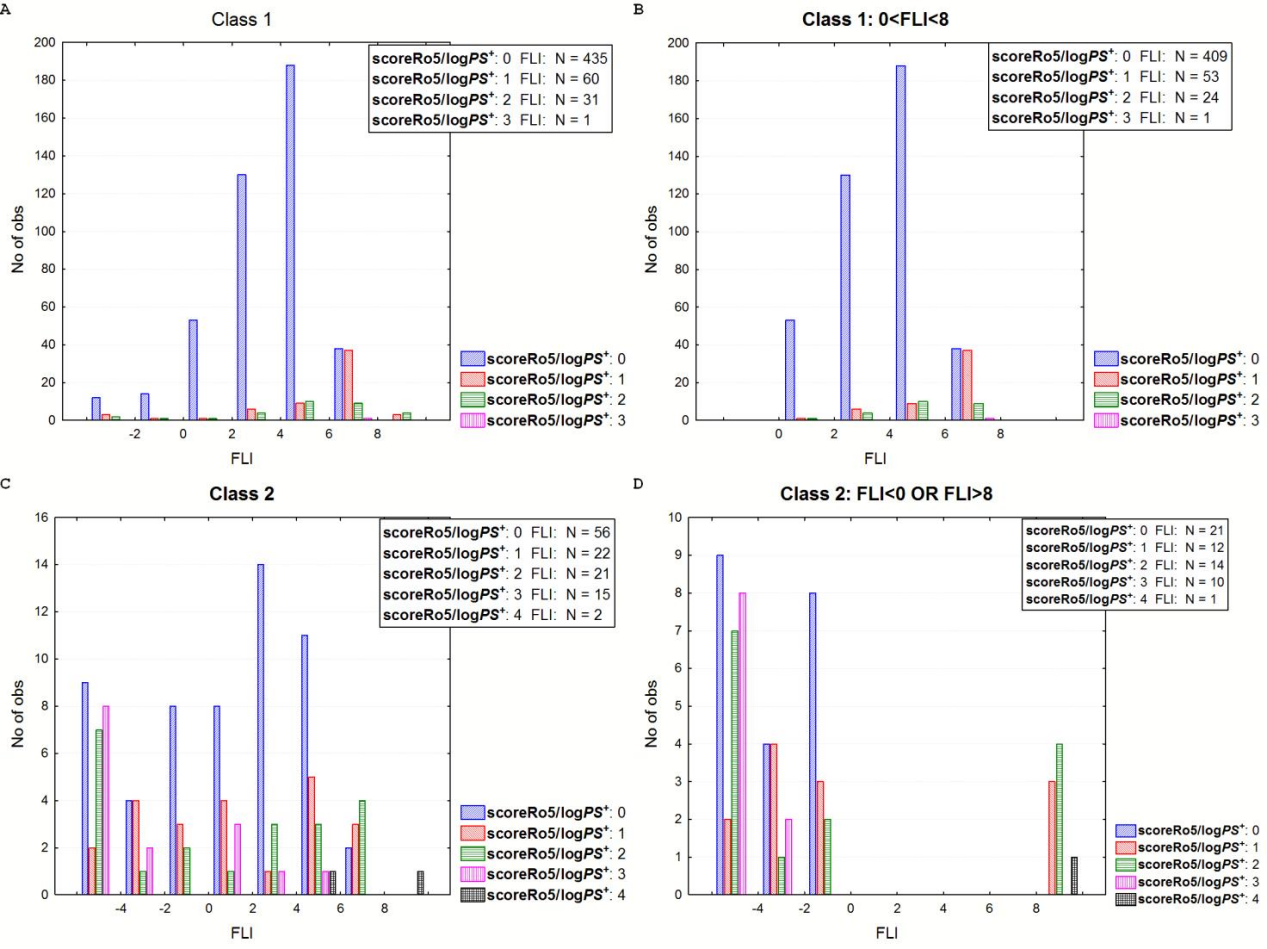
Rule of 5 violations according to FLI levels. **A**: Class 1, **B**: Class 1 subset- drugs inside the drug-like FLI range, **C**: Class 2, **D**: Class 2, subset- drugs outside the drug-like FLI range.

**Figure 11.**
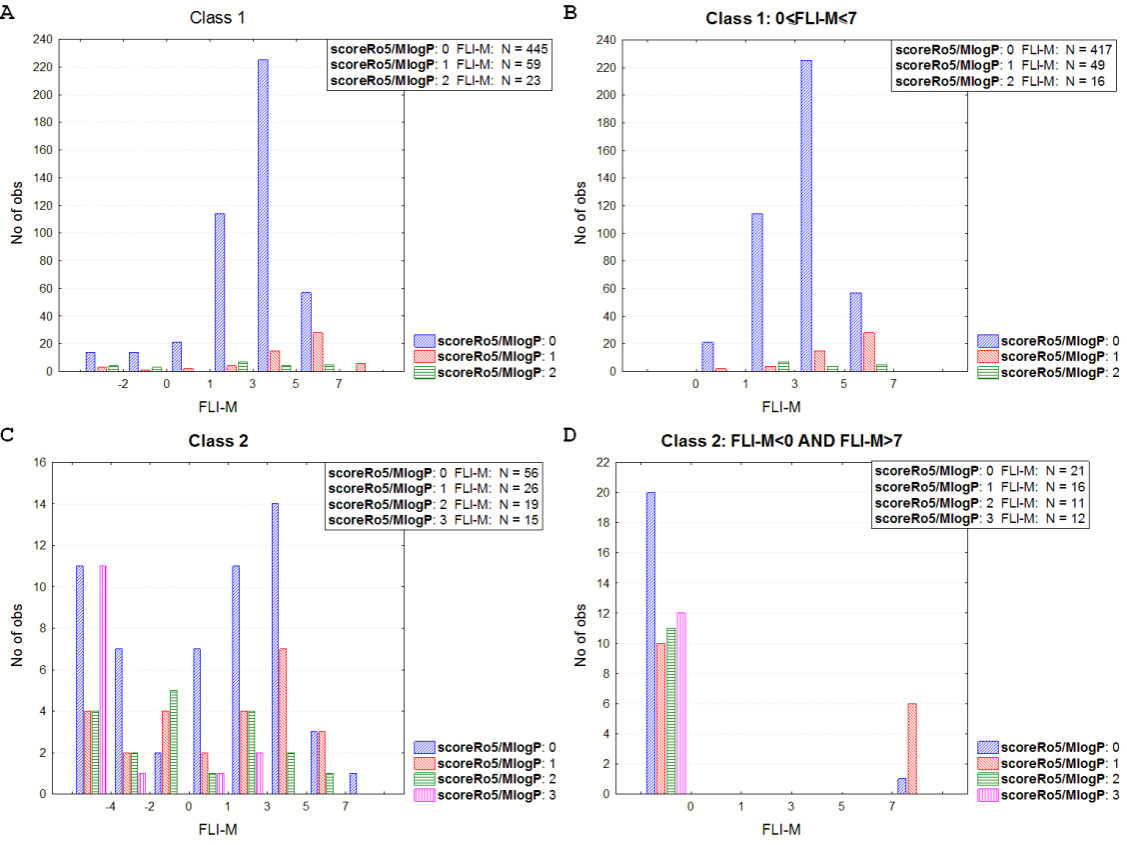
Rule of 5 violations according to FLI-M levels. **A**: Class 1, **B**: Class 1 subset- drugs inside the drug-like FLI-M range, **C**: Class 2, **D**: Class 2, subset- drugs outside the drug-like FLI-M range.

In Figure 10, the combination of FLI and Rule of five (scoreRo5/logPS^+^) is shown for class 1 and the subset, including FLI values inside the drug-like range for class 2 and the subset including drugs outside the drug-like range. It is shown that 25 drugs (5.2 %) in class 1 drug-like subset (Figure 10B) display more than twofold Ro5 violation, 12 out of them involving logPS^+^ and *M*w. Considering the FLI metric, these 12 drugs would not receive a second alert, and thus they would not be considered bRo5 drugs. On the other hand, 33 drugs (28 %) of class 2 (Figure 10D) show none or one Ro5 violation. However, in terms of their FLI values, a warning should be set for these drugs according to this metric. Considering FLI-M, 16 class 1 drugs within the drug-like range (Figure 11B) show the twofold violation of the corresponding Ro5 score, 5 of them involving MlogP >4.15. On the other hand, there should be a warning for 37 drugs of class 2 outside the drug-like range, although they show 0 or 1 Ro5 violation (Figure 11D).

**Figure 12.**
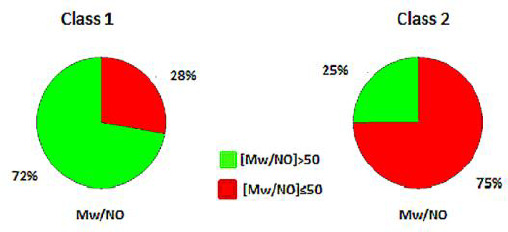
Pie charts of the distribution of Mw/NO for class 1 and class 2 drugs.

Considering the merits of FLI as a composite metric and in the light of the combination mentioned above of high *M*w with high polarity as a crucial issue for low absorption, we go a step further to propose the normalization of *M*w to the number of polar atoms [N+O]. The term *M*w/NO is calculated according to the expression *M*w/(1+[N+O]), where 1 stands to avoid division by 0 in the case of compounds that lack N and O atoms. Considering the distribution of *M*w/NO in classes 1 and 2 (Figure not shown), a discriminant value of 50 is set to differentiate the two classes. For class 1, the majority (72 %) of drugs display *M*w/NO>50, while the opposite occurs for class 2 drugs, with 75 % showing *M*w/NO≤50 (Figure 12).

## Conclusions

The previously established drug-like FLI range 0-8 is confirmed using an extended dataset, covering 93 % of highly to moderately absorbed drugs. Application of three different methods, MedChem Designer, ClogP and MlogP for lipophilicity assessment in FLI generation, supported the robustness of the results while showing that the metric is overall independent of log *P* calculation system. However, the FLI-M values generated by MlogP, which is associated with a lower drug-like upper limit, shape a narrower drug-like range between 0-7, covering 91 % of class 1 drugs. FLI tolerates higher log *P* values for drugs, provided that ionization is limited. It offers the option to explore a wider chemical space for drug discovery, notwithstanding the disadvantages of high lipophilicity regarding other issues, like promiscuity, extensive metabolism or toxicity. On the other hand, negative FLI values minimize the chances for oral absorption. The merit of FLI lies in the fact that it is a combined metric of lipophilicity and ionization and provides more information on drug characteristics while ‘softening’ the hard limits. We, therefore, suggest that a combination of metrics may be preferable to single properties. In this sense and considering the crucial two-fold violation including *M*w and [N+O], we propose a combined metric of *M*w and the number of polar atoms [*M*w/NO] to account for both size and polarity. Exploration of *M*w/NO will follow in future investigations.
