# Supplementary material for: Drug-like Properties and Fraction Lipophilicity Index as a combined metric
Source: ADMET DMPK. 2021 Oct 10;9(3):177–90. doi: 10.5599/admet.1022 (PMC8920096; doi:10.5599/admet.1022)
Supplement: Supplementary file 1 [file admet-9-1022-S1.pdf]

Supplementary tables

Drug-like Properties and Fraction Lipophilicity Index as a combined metric
Anna Tsantili-Kakoulidou, Vassilis Demopoulos
ADMET and DMPK, Vol. 9 No. 3 (2021), 177-190
https://doi.org/10.5599/admet.1022

Table 1. Descriptive statistics of drug-like properties

The entire data set				
Physicochemical Properties	N	Mean	Percentile 10	Percentile 90
log*PS*+/clog*P*/Mlog*P*	643	2.63/2.68/2.02	0.03/0.06/-0.16	5.02/5.26/3.88
log*PS*+/clog*P*/Mlog*P* (after 2000)	155	3.34/3.42/2.45	1.19/0.90/0.97	5.18/5.89/3.89
log*PS*+/clog*P*/ Mlog*P* (2010-2020)	102	3.32/3.45/2.31	1.17/0.44/0.85	5.17/6.05/3.77
log*PS*+/clog*P*/ Mlog*P* (before 2000)	488	2.40/2.44/1.88	-0.26/-0.24/-0.37	4.93/5.11/3.88
log*DS*+/clog*D*/Mlog*D**	643	1.36/1.39/0.73	-1.29/-1.53/-1.47	3.82/4.18/2.84
Mw	643	372.5	232.2	525.6
Mw (after 2000)	155	430.9	293.7	578.6
Mw (2010-2020)	102	465.1	307.5	591.5
Mw (before 2000)	488	353.5	225.3	478.6
HD/[N+O]	643	2.4/5.9	0/2	4/10
HD/[N+O](after 2000)	155	2.2/6.7	1/3	4/11
HD/[N+O](before 2000)	488	2.5/5.7	0/2	5/10
Class 1				
log*PS*+/clog*P*/*MlogP*	527	2.98/3.08/2.38	0.88/0.63/0.69	5.09/5.36/3.98
log*DS*+/clog*D*/Mlog*D**	527	1.77/1.86/1.16	-0.37/-0.57/-0.66	3.96/ 4.28/2.88
Δ(log*P*-log*D**)	527	1.23	0.28	2.11
Mw	527	348.5	230.1	481.5
HD/[N+O]	527	1.8/5.0	0/2	4/8
Class 2				
log*PS*+/clog*P*/Mlog*P*	116	1.01/0.85/0.34	-1.79/-3.14/-3.49	4.23/4.62/3.36
log*DS*+/clog*D*/Mlog*D**	116	-0.53/-0.72/-1.22	-3.71/-5.15/-5.68	3.25/3.23//1.95
Δ(log*P*-log*D**)**	115*	1.52	0.36	2.67
Mw	116	481.4	244.9	668.7
HD/[N+O]	116	5.15/10.13	2/4	15/18

*at pH 7.4 for bases and 5.5 for acids

** case 638 (Table 1S, Supplementary Material) with unrealistic value not included

Table 2: Ionization and polarity dependence in regard to lipophilicity and molecular weight for class 1 and class 2 drugs

	max. Δ(log*P*-log*D*)	max. [N+O]
Class 1:<br>logPS^{+}≤5/logPS^{+}>5<br>n=468 / n=59	3.49/2.69	15/14
Class 2:<br>logPS^{+}≤5 / logPS^{+}>5<br>n=108 / n=7*	4.89 /1.78	33/29
Class 1:<br>Mw≤600 / Mw>600<br>n=517 / n=10	3.49 /1.75	15/15
Class 2:<br>Mw≤600 / Mw>600<br>n=99 / n=16*	4.89/2.31	19/33

*case 368 excluded due to unrealistic Δ(log*P*-log*D*) value

Table 3. Violations of drug-like properties (combinations with zero cases not included)

Property	No of cases	Cases with low %FA
	1-fold violation	
logPS^+/clog$P$/Mlog$P$≤-0.4	20/18/23	11 (55%) /8(44.4%) /14(61%)
logPS^+/clog$P$/Mlog$P$ ≤-1	12/11/13	8 (66.7%) /6(54.5%)/10(77%)
logPS^+/clog$P$>5/Mlog$P$>4.15	44/42/33	2 (4.5%)/2 (4.8%)/2 (6.1%)
MW>500	16	5 (31%)
HD>5	10	6 (60%)
[N+O]>10	12	9 (75%)
	2-fold violation	
logPS^+/clog$P$/ Mlog$P$≤-0.4, [N+O]>10	5/3/2	4 (80%) /3(100%)/1 (50%)
logPS^+/clog$P$/ Mlog$P$ ≤-0.4, HD>5	4/5/5	2 (50) /2 (40%)/2 (40%)
logPS^+/clog$P$/ Mlog$P$≤-1, [N+O]>10	2/2/0	1 /2 /0
logPS^+/clog$P$<-1/ Mlog$P$≤-1, HD>5	2/2/2	1/1/2
logPS^+/clog$P$>5/Mlog$P$>*4.15*, Mw>500	20/17/7	4 (20%)/ 3 (17.6 %)/ 7 (100%)
Mw>500, [N+O]>10	26	13 (50%)
HD>5, [N+O]>10	6	4 (66.7%)
	3-fold violation	
logPS^+/clog$P$/Mlog$P$≤-0.4 Mw>500, [N+O]>10	5/6/3	4(80%) /5 (83%)/2 (66.7%)
logPS^+/clog$P$//Mlog$P$ ≤-0.4 HD>5, HA>10	5/5/4	4(80%) /4(80%)/4 (100%)
logPS^+/clog$P$/Mlog$P$≤-1, Mw>500, [N+O]>10	2/2/0	2/2/0
logPS^+/clog$P$/Mlog$P$ ≤-1, HD>5, [N+O]>10	5/5/4	4(80%)/4(80%)/4 (100%)
Mw>500, HD>5, [N+O]>10	14/15*	14 /15
logPS^+/clog$P$>5, Mw>500, [N+O]>10	1/3/0	0/0/0
	4-fold violation	
logPS^+/clog$P$≤-0.4 Mw>500, HD>5, [N+O]>10	8/8/13	8 / 8/13
logPS^+/clog$P$≤-1 Mw>500, HD>5, [N+O]>10	8/9/12	8/9/12
logPS^+/clog$P$>5 Mw>500, HD>5, [N+O]>10	2/1*/0	2/1

*case 638 (Table 1S (Supplementary Material) with logPS^+ >5 and clog$P$≤5 shows 4-fold violation based on logPS^+ and 3-fold violation based on clog$P$, respectively.
